# The Zn(II)-1,4,7-Trimethyl-1,4,7-Triazacyclononane Complex: A Monometallic Catalyst Active in Two Protonation States

**DOI:** 10.3389/fchem.2019.00469

**Published:** 2019-07-03

**Authors:** Marta Diez-Castellnou, Giovanni Salassa, Fabrizio Mancin, Paolo Scrimin

**Affiliations:** ^1^EaStCHEM School of Chemistry, University of St Andrews, St. Andrews, United Kingdom; ^2^Département de Chimie Physique, Université de Genève, Genève, Switzerland; ^3^Dipartimento di Scienze Chimiche, Università di Padova, Padova, Italy

**Keywords:** RNA, monometallic Zn(II)-complexes, azacrown, phosphate cleavage, phosphoesterase models, kinetics

## Abstract

In this paper, the unusual reactivity of the complex Zn(II)-1,4,7-trimethyl-1, 4,7-triazacyclononane (**2**) in the transesterification of the RNA-model substrate, *HPNP* (**3**), is reported. The dependence of the reactivity (k_2_) with pH does not follow the characteristic bell-shape profile typical of complexes with penta-coordinated metal centers. By the contrary, two reactive species, featuring different deprotonation states, are present, with the tri-aqua complex being more reactive than the mono-hydroxy-diaqua one. Apparently, such a difference arises from the total complex charge which plays an important role in the stability of the transition state/s of the reactions. Relevant insight on the reaction mechanism were hence obtained.

## Introduction

Phosphate diesters have a fundamental importance in the chemistry of life in particular because they constitute the backbone of essential biomolecules as DNA and RNA. Their hydrolytic stability is very high. When the sole P-O cleavage by water is considered, the half-life of DNA is estimated to be in the order of magnitude of millions of years, at 25°C and pH 7, and that of RNA is around a hundred years (Wolfenden et al., 1998; Schroeder et al., 2006). Still, the hydrolytic processing of nucleic acids occurs in living organism in few milliseconds, thanks to the enzymes devoted to this task, as nucleases and phosphatases (Westheimer, 1987; Kamerlin et al., 2013). Most of them contain metal ions, as Mg(II), Ca(II), and Zn(II), in their active sites.

In the attempt to reproduce the activity, and possibly the proficiency of enzymes, chemists have focused their attention on the creation of artificial nucleases (Morrow et al., 2008; Aiba et al., 2011; Lassila et al., 2011; Lönnberg, 2011; Mancin et al., 2012; Diez-Castellnou et al., 2017a). Despite the significant effort invested in understanding the mechanism of the enzyme catalyzed reaction (Korhonen et al., 2013; Erxleben, 2019) and, therefore, in creating efficient artificial hydrolytic agents, the enzyme reactivity is still unrivaled and several questions remain to be addressed.

A better understanding of the roles played by the metal ion in promoting the hydrolytic cleavage of phosphate esters could be achieved using simple mono- or bi-metallic complexes as models. In particular Zn(II), while not being Nature's first choice, is likely the metal ion most widely employed in artificial systems (Mancin and Tecilla, 2007). This is due to several reasons: (i) the possibility to produce well defined and relatively stable complexes with neutral ligands; (ii) the absence of a relevant redox chemistry; (iii) a good Lewis acid acidity; (iv) a modest field effect that allow the easy reorganization of the ligand shell to match the ligand or reaction requirements.

Design and investigation of bimetallic systems is often difficult since the possible formation of μ-hydroxo bridges may affect and even cancel their reactivity (Mancin and Tecilla, 2007). Mono-metallic Zn(II) complexes, on the other hand, produce usually modest rate accelerations when compared to the corresponding bimetallic ones. Still, they allow more detailed investigations of the cleavage reaction providing a simple and better defined model (Bonfá et al., 2003).

**Scheme 1:**
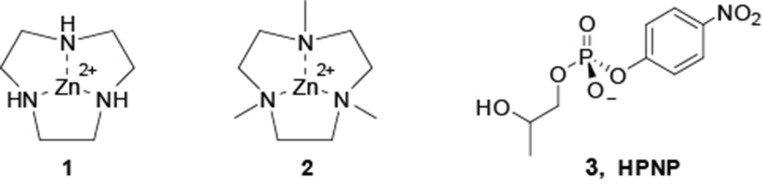
Zn(II) metal complexes used in this work and RNA-model substrate, HPNP.

In this perspective, we report here a detailed investigation of the reactivity of the Zn(II) complexes of 1,4,7-triazacyclononane (**1**) and of its methyl derivative 1,4,7-trimethyl-1,4,7-triazacyclo-nonane (**2**) toward the hydrolysis of the RNA model 2-hydroxypropyl-p-nitrophenylphosphate (*HPNP*, **3**) (see Scheme 1). We found that the reactivity of **2** follows an unprecedented behavior featuring three pH dependent reactivity breaks, providing new insight on the reactivity of these systems.

## Results

HPNP cleavage rate in the presence of the two Zn(II) complexes was measured at 40°C. Kinetic experiments were performed in pseudo-first order conditions (i.e., in the presence of an excess of metal complex over the substrate, whose concentration was fixed at 20 μM) by monitoring the formation of 4-nitrophenolate by its UV-Vis absorption at 400 nm.

The complexes were prepared *in situ* by mixing a solution of Zn(NO_3_)_2_ with the corresponding ligand, in buffer. TACN and his derivatives have a high affinity for Zn(II). Indeed, the values reported in literature for the complex formation constants assure that, in the experimental condition here used, the complexes are fully formed (Yang and Zompa, 1976).

With both the complexes **1** and **2**, the pseudo-firsts order rate constants (*k*_obs_) measured increase linearly with the Zn(II) complex concentration in the interval investigated (0–0.4 mM). Apparent second order rate constants (i.e., *k*_2_ = *k*_cat_/K_M_ in the Michaelis-Menten formalism) can be obtained by linear regression fitting of the *k*_obs_ vs. [complex] data.

**Figure 1:**
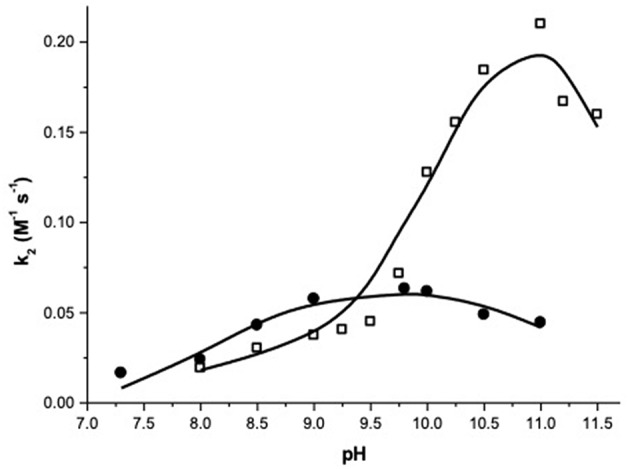
pH dependence of the second order rate constants (k_2_) for the transesterification of HPNP catalyzed by complex 1 (•) and 2 (□) at 40°C.

Figure 1 reports the pH dependence of the apparent second order rate constants measured for the two complexes. As expected, the pH has a strong influence on the reaction rates suggesting, as generally observed with similar systems, that the deprotonation of metal bound-species has a relevant role in the reaction.

At a first sight, both the profiles show the bell-shape characteristic of metal complexes with two metal bound water molecules. Indeed, kinetic data for complex **1** were fitted with Equation (1), which accounts for the reactivity of a species with three protonation states where only the second one is active (Supplementary Material). The fit provides the pK_a_ values of 8.13 and 11.28, respectively for the first and second deprotonation. The second order rate constant (k_*Zn*_) for the reaction of the active mono-deprotonated catalyst's species with the substrate is 0.064 M^−1^ s^−1^.

The values of p${\text{K}}_{\text{a}}^{1}$ and k_*Zn*_ obtained are in line with those reported in literature for complex **1** (Bonfá et al., 2003). The formation of the bis-deprotonated species and the consequent drop of the catalysts reactivity at higher pH have never been reported for **1**, but a similar behavior is well-known for similar complexes of tridentate ligands, as 1,5,9-triazacyclododecane (Livieri et al., 2007).

The fitting of the kinetic data for complex **2** with Equation (1) gives very poor results (see Supplementary Information). A closer inspection of the profile of complex **2** reveals that it does not follow exactly a bell-shape. Indeed, k_2_ smoothly increases from pH 8 to pH 9.5, with a reactivity similar to that of complex **1**, suggesting that the first deprotonation is occurring in this range. When the pH reaches the value of 9.5, a stronger, reactivity increase is observed and k_2_ reaches values 4-fold larger than the maximum one reached by complex **1**. Eventually, the reactivity starts to drop above pH 11.0. A good fit of the data was obtained by using Equation (2), which was written for a reactivity model that involves a species with four protonation states and with two of them, the second and the third, reactive (Supplementary Material). Note, one of the referees pointed out that highly correlated parameters are obtained in the case of 5 variables fitting. As discussed, reliability of the reactivity and acidity parameters obtained is however supported by the comparison with the pH reactivity profile of complex **1**.

The results of the fittings are reported in Table 1. The three pK_a_ values obtained (7.7, 10.2, and 11.6) are separated respectively by 2.5 and 1.4 pK_a_ units, with the first pK_a_ value quite close to the corresponding one measured for **1**. Also the *k*_Zn_ value for the mono-deprotonated species is on the same order of magnitude to that measured for **1** (0.025 M^−1^ sec^−1^) but that of the bis-deprotonated species (*k'*_Zn_) is considerably larger (0.27 M^−1^ sec^−1^).

**Table 1:** Kinetic parameters from pH rate profiles.

**Complex**	**$pK_{a}^{1}$**	**$pK_{a}^{2}$**	**$pK_{a}^{3}$**	**k_Zn_ (M^−1^·s^−1^)**	**$k_{Zn}^{\prime }$(M^−1^·s^−1^)**
**1**	8.13	11.28	-	0.064	-
**2**	7.71	10.18	11.6	0.025	0.27

*Conditions: [HPNP] = 2.0·10^−5^ M, [buffer] = 5.0·10^−2^ M, at 40 °C*.

## Discussion

The presence of a third metal bound water molecule in complex **2** is confirmed by the crystal structure obtained by Trogler and coworkers (Silver et al., 1995) for the complex [Zn(Me_3_tacn)(H_2_O)_3_]^2+^ (**2·3H**_**2**_**O**)^**2+**^ where the Zn(II) ion adopts an octahedral environment, coordinated to three nitrogens of the macrocycle and also to three aqua ligands. In addition, Spiccia and coworkers (Fry et al., 2003) reported the crystal structure for the related complex [Zn_2_(Me_3_tacn)_2_(H_2_O)_4_(PhOPO_3_)]^2+^(**2**_2_ **·4H**_**2**_**O·PhPO**_**4**_**)**^**2+**^ where the Zn(II) ion is coordinated to three nitrogen atoms from N,N,N-trimethyl-1,4,7,-triazacyclononane, two water molecules and one oxygen from phenyl phosphate. These structures support our hypothesis that in complex **2**, formed by Zn(II) with the ligand 1,4,7-trimethyl-1,4,7-triazacyclononane, the coordination number of the metal ion is 6, as the accessible surface of the metal ion is large enough to allow coordination of two water molecules and of the substrate simultaneously. Unfortunately, no crystal structures are available for complex **1** to confirm its preference for the coordination number 5 at the metal ion, as suggested by kinetic experiments. The *flexibility* of the Zn(II) ion, which does not have a defined coordination geometry, is well-known (Sigel and Bruce Martin, 1994).

Potentiometric titration data available are also quite scarce. In the case of complex **1**, precipitation above pH 8 prevented the determination of the acidity of the metal bound water molecules. Kinetic experiments, as already mentioned, confirm a pK_a_ value around 8 for the first water molecule (Bonfá et al., 2003). In the case of complex **2**, Trogler and coworkers reported the values of 10.9 and 12.3 (Silver et al., 1995). Such values appear surprisingly high when compared with complex **1** and with other Zn(II) complexes of macrocyclic polyamides (Kimura et al., 1990; Koike et al., 1990, 1991, 1992; Kimura and Koike, 1991). Indeed, one would expect that electron-donating methyl group will lower the acidity of the substituted metallic complex with respect to the unsubstituted one (Canary et al., 1995). Interestingly, the similarity of these values with those here reported for the second and third pK_a_ is clear (Fry et al., 2005).

**Scheme 2:**
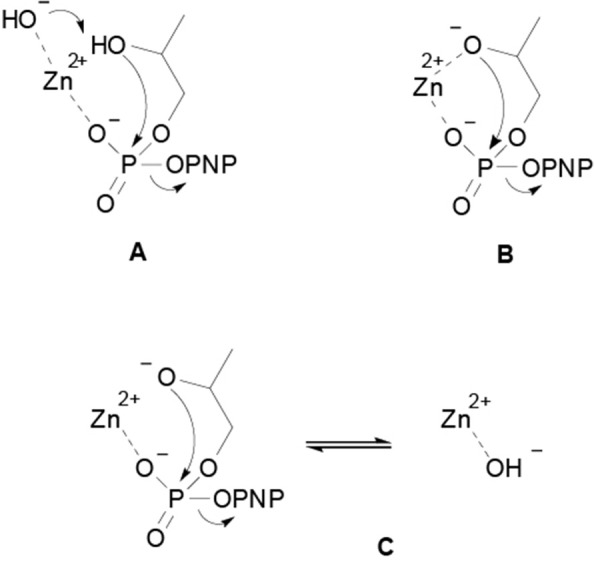
Proposed mechanisms for the metal catalyzed HPNP transesterification reaction: **(A)** the substrate deprotonation by a metal bound hydroxide occurs simultaneously to the nucleophilic attack on the phosphorus atom; **(B)** the substrate alcoholic group is bound to the metal ion and deprotonates before the nucleophilic attack; **(C)** the substrate deprotonation occurs before metal coordination and the deprotonation of a metal bound water molecule decreases the interaction of metal with the substrate.

Focusing the attention on the hydrolytic reactivity of the two complexes, it is relevant to note that the pH profiles obtained confirm the coordination of the substrate to the complex. No other possible mechanism, as general base catalysis or nucleophile catalysis, would account for the reactivity decrease observed at high pH values. There are three kinetically equivalent mechanisms that have been proposed to explain the pH dependence observed for the metal catalyzed HPNP transesterification (Korhonen et al., 2013; Diez-Castellnou et al., 2017a). In the first, the reaction involves the deprotonation of the substrate's hydroxyl group by a metal bound hydroxide, which acts as a general base, simultaneously to the nucleophilic attack on the phosphorus atom (Scheme 2A). In the second, the substrate alcoholic group is bound to the metal ion and deprotonates before the nucleophilic attack (Scheme 2B). This mechanism is indistinguishable from the others because the pK_a_ values of metal bound water molecules and alcoholic hydroxyls are similar (Kimura et al., 1995; Livieri et al., 2007). In the third mechanism (Scheme 2C) the substrate deprotonation occurs before metal coordination and the deprotonation of a metal bound water molecules decreases (or hamper) the interaction of the metal with the substrate.

Several experimental evidences strongly support the last mechanism (2C) in the case of catalysts that have coordination sites available on the metal ion to accommodate only the substrate (Morrow et al., 2008; Korhonen et al., 2013; Diez-Castellnou et al., 2017a). On the other hand, it is quite likely that when the catalyst have more coordination sites, the substrate alkoxide will bind to the metal ion, turning the reaction mechanism into path 2B (Livieri et al., 2007). In this mechanism, the metal ion plays two opposite roles: (i) it increases the reactivity of the substrate toward the nucleophilic attack, acting as a Lewis acid, (ii) it decreases the reactivity of the nucleophile, by decreasing its pK_a_.

**Figure 2:**
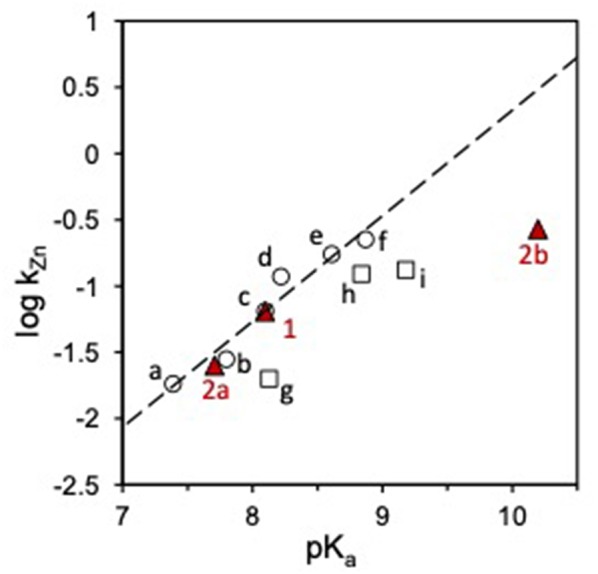
Plot of log *k*_Zn_ vs. *p*K_*a*_ for the transesterification of HPNP catalyzed by Zn(II) complexes in water. Red triangles represent the reactivity of complexes **1** and **2**, circles (○) are cyclic triamine ligands (a-f), squares (□) linear triamine ligands (g-i), see ref Bonfá et al. (2003) and Bonomi et al. (2013). The dashed line shows the linear fit of the reactivity data for the complexes of cyclic triamines (slope = 0.80). The reactivity of **1** and **2** was corrected to account for the temperature difference by matching the reactivity of **1** with the reported data (ref. Bonfá et al., 2003).

A few years ago, we calculated the *k*_Zn_ values for the HPNP cleavage promoted by several mononuclear Zn(II) complexes of polyamine ligands (Figure 2) (Bonfá et al., 2003; Bonomi et al., 2013). In the case of triamine complexes, we found a positive correlation of the reactivity with the pK_a_ of the first deprotonation of a metal bound species. Such a behavior suggested that, among the two concomitant and counterbalancing effects active, the loss of reactivity of the nucleophile prevails over the Lewis acid activation of the substrate.

**Scheme 3:**
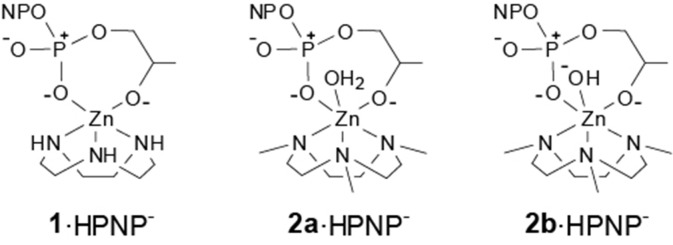
Proposed structure of the pre-reactive complexes of 1 and 2 with HPNP.

When the reactivity of complexes **1** and **2** is analyzed in this perspective, interesting insight is provided. The reactivity of complexes **1** and **2a** (Scheme 3) is in line with that of other triamine complexes (Figure 2). On the other hand, the reactivity of mono-deprotonated complex **2b** is about 10 times smaller than that expected for a complex of a triamine ligand with that pK_a_. This suggest that a further effect is at play in this case besides substrate Lewis acid activation and nucleophile reactivity modulation. Significant modifications of the coordination geometries in the two complexes **2a** and **2b**, which could justify their different reactivity, are unlikely as confirmed by DFT optimization of the complex geometries (see Supplementary Material). The other relevant differences between the reactive protonation states of complex **2** are essentially two. First, in complex **2a** there is a metal bound water molecule, which could provide intramolecular H-bonds or acid catalysis. Since this water molecule is turned into an hydroxide in **2b**, its positive effect on reactivity would be canceled. If this was the case (or if a general base catalysis mechanism was active), however, reactivity of **2a** would be greater than that of **1** and of the other triamine complexes. The second difference is in the total charge of the reactive species, which is 0 in the ternary complex of **2a** with HPNP and−1 in the case of **2b**. Upon the nucleophilic attack of the substrate alkoxide, additional negative charge builds up in the phosphoryl group and this is electrostatically disfavored by the overall complex charge, resulting into a lower reactivity. Indeed, we earlier reported as the insertion of an additional positive charge in the ligand structure, located in close proximity to the reaction site, can increase the reactivity of a mononuclear Zn(II) complex up to one order of magnitude. This is in good agreement with the 10-fold rate decrease estimated for complex **2b** (Bonomi et al., 2009).

## Conclusions

In conclusion, in this paper we have demonstrated as even small modifications in the structure of the ligands, as the insertion of methyl groups in the nitrogen atoms of triazacyclononane, may have a relevant effect on the ability of the corresponding Zn(II) complex in promoting the phosphoryl transfer reaction. The complex **2** is, at the best of our knowledge, the only monometallic Zn(II) catalyst of HPNP cleavage that is active in two different protonation states. Such a reactivity is due to the peculiar ability of 1,4,7-trimethyl-1,4,7-triazacyclononane to increase the number of coordination sites to 6 on the metal ion with respect of other similar ligands. The most relevant result of this study is however the mechanistic information obtained. The reactivity of complex **2** further supports mechanism 2B as the most likely in the case of complexes where enough solvent-occupied coordination sites on the metal are available. It also confirms that hydrolytic reactivity of these systems is the result of a delicate counterbalance between Lewis acid and electrostatic activation of the substrate (and stabilization of the transition state) and the decrease of the nucleophile reactivity. Several evidences obtained with model systems, and confirmed by our results, suggest that optimum reactivity should be obtained when the nucleophile is not bound to the metal ion and the overall complex charge is as great as possible (Tirel et al., 2014; Tirel and Williams, 2015). This explains also the greater reactivity usually observed with trivalent cations, as well as the detrimental effect of negatively charged ligands (Mancin et al., 2012). Metal ion coordination of the nucleophile may however be necessary to increase the fraction of nucleophile available at physiological pH and to provide a better preorganization of the latter for the attack to the phosphorous atom. The need to consider of all these factors explains the difficulties in reproducing enzymes' proficiency with simple models. Indeed only a precise spatial organization of multiple active species can optimize the positive effects while minimizing the detrimental one. It is hence not surprising the fact that only by using sophisticated supramolecular architectures, where several beneficial factors can be implemented, remarkable reactivities were obtained (Manea et al., 2004; Feng et al., 2006; Bonomi et al., 2008; Gruber et al., 2011; Diez-Castellnou et al., 2014).

### Equations

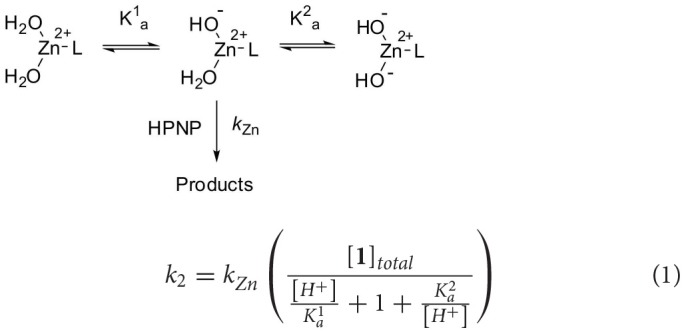

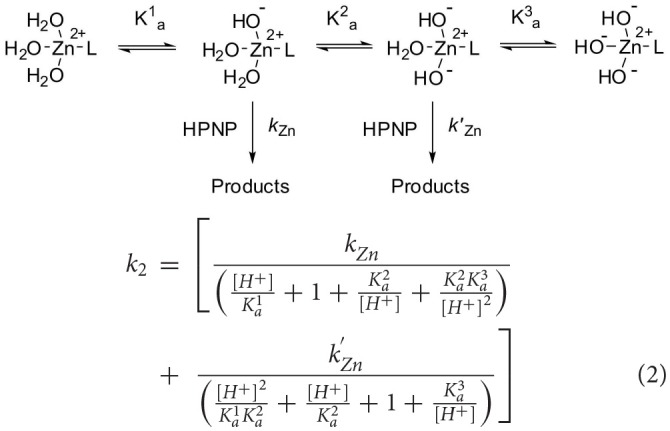





## Data Availability

All datasets generated for this study are included in the manuscript and/or the Supplementary Files.

## Author Contributions

MD-C contributed conception and design of the present work, collected and analyzed the kinetic data, and wrote the manuscript. GS performed the DFT calculations. FM and PS supervised this project. All authors contributed to manuscript revision, read, and approved the submitted version.

### Conflict of Interest Statement

The authors declare that the research was conducted in the absence of any commercial or financial relationships that could be construed as a potential conflict of interest.

## Supplementary Material

DFT calculations and additional kinetic experiments.

## Abbreviations

HPNP: 2-hydroxypropyl-p-nitrophenylphosphate.
