# Supplementary material for: The Zn(II)-1,4,7-Trimethyl-1,4,7-Triazacyclononane Complex: A Monometallic Catalyst Active in Two Protonation States
Source: Front Chem. 2019 Jul 3;7:469. doi: 10.3389/fchem.2019.00469 (PMC6616306; doi:10.3389/fchem.2019.00469)
Supplement: Supplementary file 1 [file Data_Sheet_1.PDF]

Supplementary Material

The Zn(II)-1,4,7-trimethyl-1,4,7-triazacyclononane complex: a monometallic catalyst active in two protonation states.

Marta Diez-Castellnou, Giovanni Salassa, Fabrizio Mancin, Paolo Scrimin

Table of Contents

1. Experimental procedures 2

2. Values of the second order rate constants 4

3. Kinetics equations 5

    3.1. Complex **1** 5

    3.2. Complex **2** 7

4. DFT calculations 11

    4.1. DFT minimized structures 11

    4.2. XYZ coordinates of minimized structures 13

1. Experimental Procedures.

General: Ligands **1** and **2** are commercially available and were used as received.

UV-Visible spectra and kinetic traces were recorded on Cary 50 spectrophotometer equipped with thermostated multiple cell holders.

$Zn(NO_3)_2$ was an analytical grade product. Metal ion concentration in stock solutions were measured by atomic absorption spectroscopy using Perkin Elmer 1100 instrument.

Buffers used for kinetic experiments were used as supplied by the manufacturers: 2-morpholinoethanesulfonic acid (MES, Fluka), 4-(2-hydroxyethyl)-1-piperazineethanesulfonic acid (HEPES, Sigma), 4-(2-hydroxyethyl)-1-piperazinepropanesulfonic acid (EPPS, Sigma), 2-[N-cyclohexylamino]ethanesulfonic acid (CHES, Sigma), 3-[cyclohexylamino]1-propanesulfonic acid (CAPS, Sigma), piperidine (Sigma).

The substrate 2-hydroxylpropyl-4-nitrophenyl phosphate (HPNP) was synthesized as described.[i]

Kinetic Measurements: The reactions were started by adding 20 μL of a 1 mM water solution of substrate (HPNP) to a 1 mL solution containing the appropriate buffer (0.05 M), increasing amounts of $Zn(NO_3)_2$ and of triazacyclononane (TACN) or trimethyltriazacyclononane (TMTACN) ligand. Substrate conversion was monitored by following the absorption of p-nitrophenoxide at 400 nm using a Varian Cary 50 Spectrophotometer equipped with a thermostat cell-holder (Figure S1). Pseudo-first order rate constants were obtained by fitting the absorbance versus time data to the following equation 3:

$$Abs = A1 - A2 * e^{-k_w * x} \qquad (3)$$

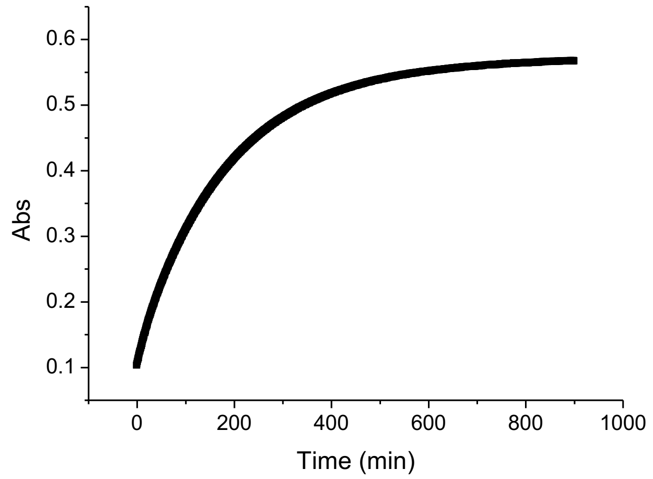

Figure S1. Example of the increase of absorbance versus time. Conditions: [HPNP] = 2.0 × 10^{-5} M, [buffer] = 5.0 × 10^{-2} M, at 40°C

Pseudo-first order rate constants have a linear dependence with the catalyst concentration (Figure S2) and allow us to obtain the second order rate constant of the reaction by fitting the data to equation 4:

$$k_w = k_2[cat] + a \qquad (4)$$

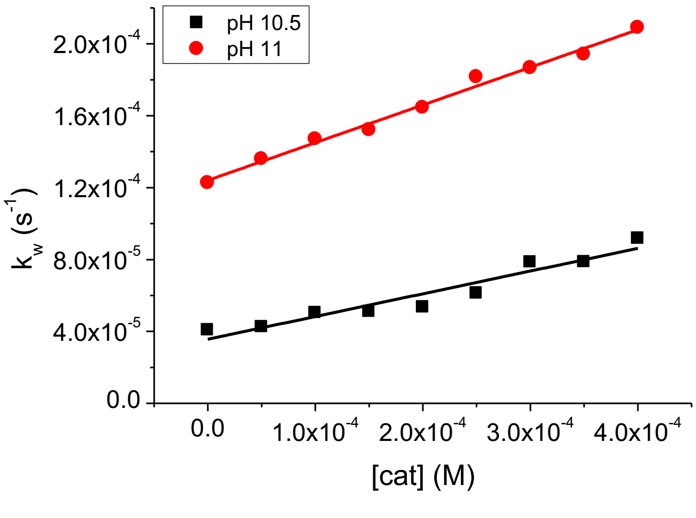

Figure S2. Example of the linear dependence of the pseudo-first order rate constants with the concentration of catalyst **2** at 40°C.

2. Values of the second order rate constants.

The following Table 2 contains the values of the second order rate constants obtained for complex **1** and **2** at different pHs and 40 °C. The data here reported were used to plot Figure 1.

Table 1. Second order rate constant values for the HPNP cleavage in the presence of complex **1** or complex **2** at 40 °C.

**pH**	k_2[a] (M^{-1} s^{-1})	k_2[b] (M^{-1} s^{-1})
7.30	0.0165 ± 0.0007	-
8.00	0.0244 ± 0.0016	0.0192 ± 0.0027
8.50	0.0430 ± 0.0056	0.0302 ± 0.0036
9.00	0.0575 ± 0.0129	0.0374 ± 0.0048
9.25	-	0.0406 ± 0.0041
9.50	-	0.0449 ± 0.0030
9.80	0.0632 ± 0.0078	0.0716 ± 0.0040
10.00	0.0616 ± 0.0054	0.1277 ± 0.0050
10.25	-	0.1554 ± 0.0072
10.50	0.0488 ± 0.0052	0.1843 ± 0.0187
11.00	0.0444 ± 0.0060	0.2099 ± 0.0117
11.20	-	0.1670 ± 0.0129
11.50	-	0.1597 ± 0.0161

a) Values for complex **1**. b) Values for complex **2**. (Conditions: [HPNP] = 2.0×10^{-5} M, [buffer] = 5.0×10^{-2} M).

3. Kinetics equations.

3.1. Complex 1

The mechanistic pathway of complex **1** is detailed in Figure S3. The scheme was used to obtain equation 1. Since the reactivity versus catalyst concentration plots are linear, $K_{1,2}$ and $k_{1,2}$ could not be extrapolated independently and the reaction was considered to be a second order one (with $k' = k/K$)

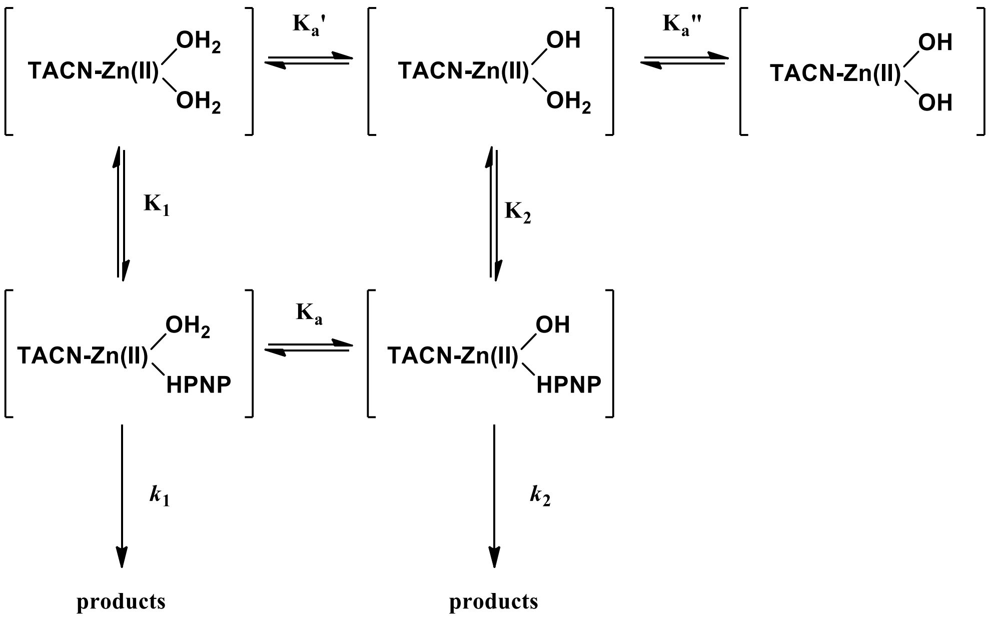

Figure S3. Scheme of the mechanism followed by the complex **1**.

Mass balances

The total amount of TACN-Zn(II) in solution is the sum of the 3 different species:

$$[TACN \cdot Zn(II)]_{total} = [TACN \cdot Zn(II) - (H_2O)_2] + [TACN \cdot Zn(II) - (OH)(H_2O)] + [TACN \cdot Zn(II) - (OH)_2] \quad (5)$$

These species are related each other by the equilibrium of deprotonation:

$$K_a^1 = \frac{[TACN{\cdot}Zn(II){-}(OH)(H_2O)][H^+]}{[TACN{\cdot}Zn(II){-}(H_2O)_2]} \quad (6) \qquad K_a^2 = \frac{[TACN{\cdot}Zn(II){-}(OH)_2][H^+]}{[TACN{\cdot}Zn(II){-}(OH)(H_2O)]} \quad (7)$$

If we include equations 6 and 7 in the mass balance 5, we obtain the equation 8 which is a relation between the total amount of TACN-Zn(II) complex towards the active catalytic species $[TACN \cdot Zn(II) - (OH)(H_2O)]$:

$$[TACN \cdot Zn(II)]_{total} = \frac{[H^+]}{K_a^1}[TACN \cdot Zn(II) - (OH)(H_2O)] + [TACN \cdot Zn(II) - (OH)(H_2O)] + \frac{K_a^2}{[H^+]}[TACN \cdot Zn(II) - (OH)(H_2O)]$$

$$[TACN \cdot Zn(II)]_{total} = \left(\frac{[H^+]}{K_a^1} + 1 + \frac{K_a^2}{[H^+]}\right)[TACN \cdot Zn(II) - (OH)(H_2O)] \quad (8)$$

On the other hand, the reaction rate observed (k_φ) is measured by the consumption of HPNP with time:

$$k_\varphi = \frac{-d[HPNP]}{dt} = k_1[TACN \cdot Zn(II) - (H_2O)(HPNP)] + k_2[TACN \cdot Zn(II) - (OH)(HPNP)] \quad (9)$$

Where $k_1 \ll k_2$ and the concentration of catalyst-substrate complex is related to the concentration of active species$[TACN \cdot Zn(II) - (OH)(H_2O)]$ by K_2:

$$k_\varphi = \frac{-d[HPNP]}{dt} = k_2(K_2[TACN \cdot Zn(II) - (OH)(H_2O)][HPNP]) \quad (10)$$

In pseudo-first order conditions: $[TACN \cdot Zn(II) - (OH)(H_2O)][HPNP] \approx [TACN \cdot Zn(II) - (OH)(H_2O)]$

$$k_\varphi = k_2'[TACN \cdot Zn(II) - (OH)(H_2O)] \quad (11)$$

Where k_2' includes the complex formation constant, K_2.

Finally, we substituted equation 8 in 11 and we obtained the relation between the observed reaction rate k_φ and the concentration of protons (equation 1). This equation allowed us to calculate the first and second *pK$_a$* of the complex TACN-Zn(II) catalyst and also the second order reaction rate between the active species and the substrate.

$$k_\varphi = k_2'\left(\frac{[TACN \cdot Zn(II)]_{total}}{\frac{[H^+]}{K_a^1} + 1 + \frac{K_a^2}{[H^+]}}\right) \quad \textbf{(1)}$$

3.2. Complex 2

The mechanistic pathway of complex **2** includes another water molecule bound to the Zn(II). Equation 2 was obtained by applying mass balance to the catalytic mechanism of cleavage of HPNP by complex Zn(II)-1,4,7-trimethyl-1,4,7-triazacyclo-nonane, *TMTACN-Zn(II)*:

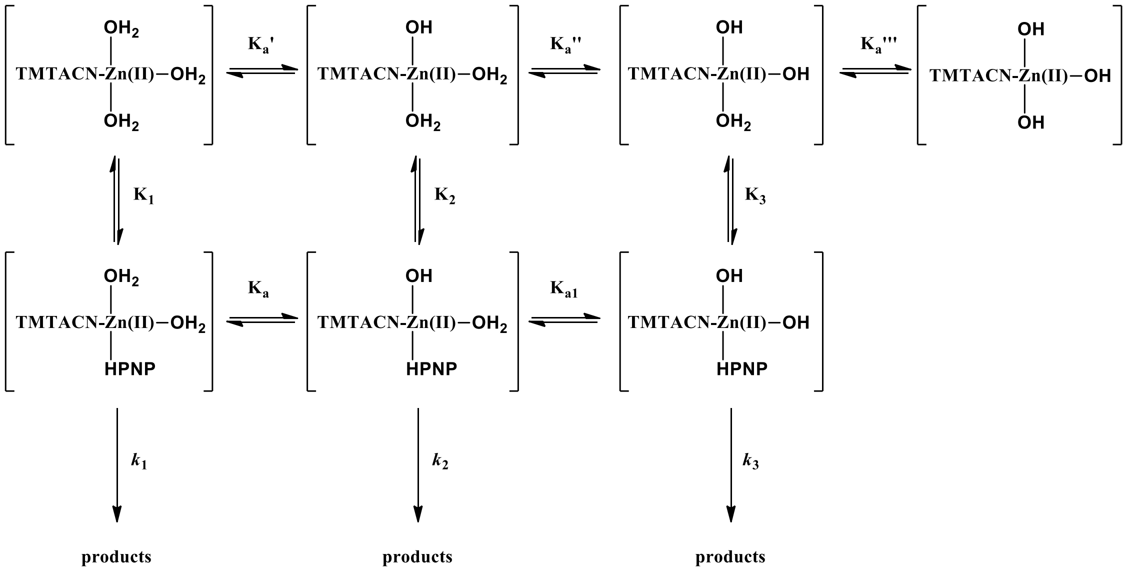

Figure S4. Scheme of the mechanism followed by the complex **2**.

Mass balances

The total amount of TMTACN-Zn(II) in solution is the sum of the 3 different species:

$$[TMTACN \cdot Zn(II)]_{total} = [TMTACN \cdot Zn(II) - (H_2O)_3] + [TMTACN \cdot Zn(II) - (OH)(H_2O)_2] + [TMTACN \cdot Zn(II) - (OH)_2(H_2O)] + [TMTACN \cdot Zn(II) - (OH)_3] \quad (12)$$

These species are related each other by the equilibria of deprotonation:

$$K_a^1 = \frac{[TMTACN{\cdot}Zn(II){-}(OH)(H_2O)_2][H^+]}{[TMTACN{\cdot}Zn(II){-}(H_2O)_3]} \quad (13) \qquad K_a^2 = \frac{[TMTACN{\cdot}Zn(II){-}(OH)_2(H_2O)][H^+]}{[TMTACN{\cdot}Zn(II){-}(OH)(H_2O)_2]} \quad (14)$$

$$K_a^3 = \frac{[TMTACN{\cdot}Zn(II){-}(OH)_3][H^+]}{[TMTACN{\cdot}Zn(II){-}(OH)_2(H_2O)]} \quad (15)$$

If we include equations 13 and 15 in the mass balance 12, we obtain the equation 16 which is a relation between the total amount of TMTACN-Zn(II) complex towards the active catalytic species $[TMTACN \cdot Zn(II) - (OH)(H_2O)_2]$:

$$[TMTACN \cdot Zn(II)]_{total}$$
$$= [TMTACN \cdot Zn(II) - (H_2O)_3] + [TMTACN \cdot Zn(II) - (OH)(H_2O)_2]$$
$$+ [TMTACN \cdot Zn(II) - (OH)_2(H_2O)] + \frac{K_a^3}{[H^+]}[TMTACN \cdot Zn(II) - (OH)_2(H_2O)]$$
$$= [TMTACN \cdot Zn(II) - (H_2O)_3] + [TMTACN \cdot Zn(II) - (OH)(H_2O)_2]$$
$$+ \left(1 + \frac{K_a^3}{[H^+]}\right)[TMTACN \cdot Zn(II) - (OH)_2(H_2O)]$$

$$[TMTACN \cdot Zn(II)]_{total}$$
$$= \frac{[H^+]}{K_a^1}[TMTACN \cdot Zn(II) - (OH)(H_2O)_2] + [TMTACN \cdot Zn(II) - (OH)(H_2O)_2]$$
$$+ \left(1 + \frac{K_a^3}{[H^+]}\right)\frac{K_a^2}{[H^+]}[TMTACN \cdot Zn(II) - (OH)(H_2O)_2]$$

$$[TMTACN \cdot Zn(II)]_{total} = \left(\frac{[H^+]}{K_a^1} + 1 + \frac{K_a^2}{[H^+]} + \frac{K_a^2 K_a^3}{[H^+]^2}\right)[TMTACN \cdot Zn(II) - (OH)(H_2O)_2] \quad (16)$$

If we include equations 14 and 15 in the mass balance 12, we obtain the equation 17 which is a relation between the total amount of TMTACN-Zn(II) complex towards the active catalytic species $[TMTACN \cdot Zn(II) - (OH)_2(H_2O)]$:

$$[TMTACN \cdot Zn(II)]_{total}$$

$$= \frac{[H^+]}{K_a^1}[TMTACN \cdot Zn(II) - (OH)(H_2O)_2] + [TMTACN \cdot Zn(II) - (OH)(H_2O)_2]$$

$$+ [TMTACN \cdot Zn(II) - (OH)_2(H_2O)] + [TMTACN \cdot Zn(II) - (OH)_3]$$

$$= \left(\frac{[H^+]}{K_a^1} + 1\right)[TMTACN \cdot Zn(II) - (OH)(H_2O)_2]$$

$$+ [TMTACN \cdot Zn(II) - (OH)_2(H_2O)] + [TMTACN \cdot Zn(II) - (OH)_3]$$

$$[TMTACN \cdot Zn(II)]_{total}$$

$$= \left(\frac{[H^+]}{K_a^1} + 1\right)\frac{[H^+]}{K_a^2}[TMTACN \cdot Zn(II) - (OH)_2(H_2O)]$$

$$+ [TMTACN \cdot Zn(II) - (OH)_2(H_2O)] + \frac{K_a^3}{[H^+]}[TMTACN \cdot Zn(II) - (OH)_2(H_2O)]$$

$$[TMTACN \cdot Zn(II)]_{total} = \left(\frac{[H^+]^2}{K_a^1 K_a^2} + \frac{[H^+]}{K_a^2} + 1 + \frac{K_a^3}{[H^+]}\right)[TMTACN \cdot Zn(II) - (OH)_2(H_2O)] \quad (17)$$

The reaction rate observed (k_φ) is measured by the consumption of HPNP with time:

$$k_\varphi = \frac{-d[HPNP]}{dt}$$

$$= k_1[TMTACN \cdot Zn(II) - (H_2O)_2(HPNP)] \quad (18)$$

$$+ k_2[TMTACN \cdot Zn(II) - (OH)(H_2O)(HPNP)]$$

$$+ k_3[TMTACN \cdot Zn(II) - (OH)_2(HPNP)]$$

Where $k_1 \ll k_2\, and\, k_3$, and the concentration of catalyst-substrate complexes is related to the concentration of active species $[TMTACN \cdot Zn(II) - (OH)(H_2O)_2]$ and $[TMTACN \cdot Zn(II) - (OH)_2(H_2O)]$ by K_2 and K_3, respectively:

$k_{\varphi} = \frac{-d[HPNP]}{dt} = k_2(K_2[TMTACN \cdot Zn(II) - (OH)(H_2O)_2][HPNP]) + k_3(K_3[TMTACN \cdot Zn(II) - (OH)_2(H_2O)][HPNP])$ (19)

In pseudo-first order conditions,

$[TMTACN \cdot Zn(II) - (OH)(H_2O)_2][HPNP] \approx [TMTACN \cdot Zn(II) - (OH)(H_2O)_2]$ and

$[TMTACN \cdot Zn(II) - (OH)_2(H_2O)][HPNP] \approx [TMTACN \cdot Zn(II) - (OH)_2(H_2O)]$:

$$k_{\varphi} = k_2'[TMTACN \cdot Zn(II) - (OH)(H_2O)_2] + k_3'[TMTACN \cdot Zn(II) - (OH)_2(H_2O)] \quad (20)$$

Where k_2' and k_3' includes the complex formation constant K_2 and K_3, respectively.

Finally, we substituted equations 16 and 17 in equation 20 and we obtained the relation between the observed reaction rate k_{φ} towards the concentration of protons (equation 2) which allowed us to calculate the first, second and third *pKa* of the complex TACN-Zn(II) catalyst and also the second order reaction rate between the active species and the substrate, k_2' and k_3'.

$$k_{\varphi} = k_2' \frac{[TMTACN \cdot Zn(II)]_{total}}{\left(\frac{[H^+]}{K_a^1} + 1 + \frac{K_a^2}{[H^+]} + \frac{K_a^2 K_a^3}{[H^+]^2}\right)} + k_3' \frac{[TMTACN \cdot Zn(II)]_{total}}{\left(\frac{[H^+]^2}{K_a^1 K_a^2} + \frac{[H^+]}{K_a^2} + 1 + \frac{K_a^3}{[H^+]}\right)}$$

$$k_{\varphi} = \left[\frac{k_2'}{\left(\frac{[H^+]}{K_a'} + 1 + \frac{K_a''}{[H^+]} + \frac{K_a'' K_a'''}{[H^+]^2}\right)} + \frac{k_3'}{\left(\frac{[H^+]^2}{K_a' K_a''} + \frac{[H^+]}{K_a''} + 1 + \frac{K_a'''}{[H^+]}\right)}\right][TMTACN \cdot Zn(II)]_{total} \quad \textbf{(2)}$$

4. DFT Calculations.

All calculations were performed with the Gaussian 09 (G09) program package[ii] employing the DFT method with hybrid functional of Truhlar and Zhao (M06)[iii], using the conductor like polarizable continuum model method (CPCM)[iv,v] with H_2O as a solvent. The LanL2DZ basis set[vi] and effective core potential were used for the Zn atom, and the split-valence 6-31G** basis set[vii] was applied for all other atoms. Geometry optimizations of the complexes were performed without any constraint, and the nature of all stationary points was confirmed by normal-mode analysis.

4.1. DFT minimized structures

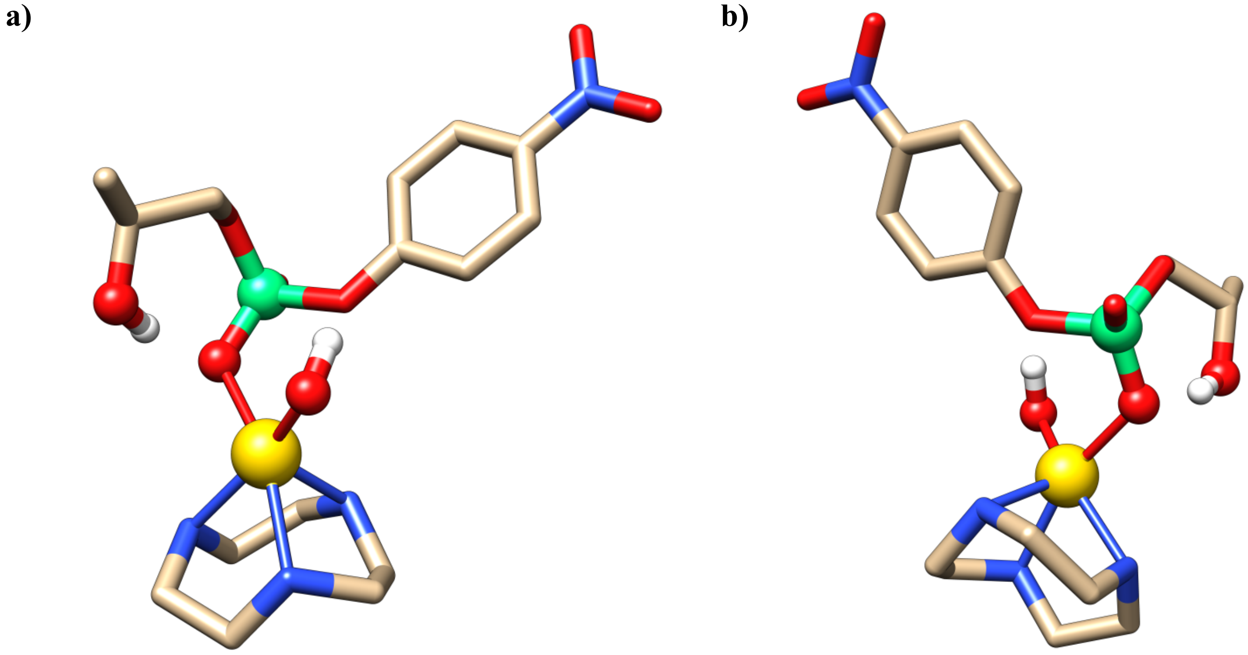

Figure S5. Two different views of DFT minimization of the structure of active species of complex **1** coordinated to the HPNP ([TACN-Zn(II)(OH)(HPNP)]). Hydrogen atoms from the ligand and the substrate have been removed for clarification, except the ones of the hydroxide groups.

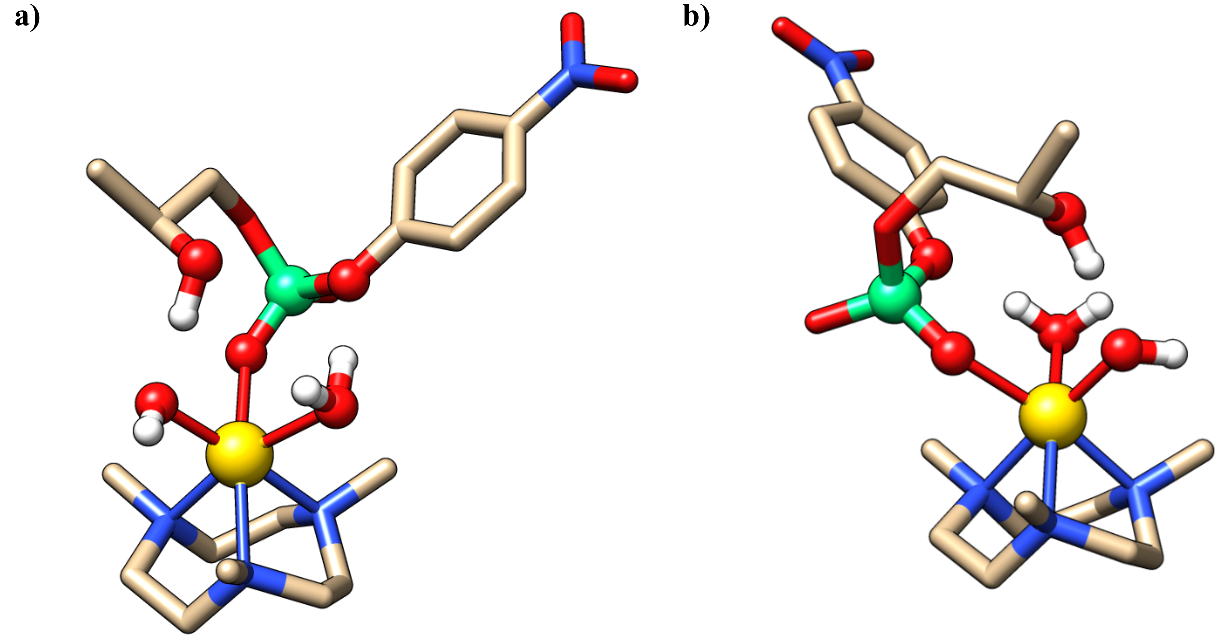

Figure S6. Two different views of the DFT minimization of the structure of the mono-deprotonated active species of complex 2 coordinated to the HPNP ([TMTACN-Zn(II)(OH)(H_2O)(HPNP)]). Hydrogen atoms from the ligand and the substrate have been removed for clarification, except the ones of the hydroxide groups and the metal-bound water molecule.

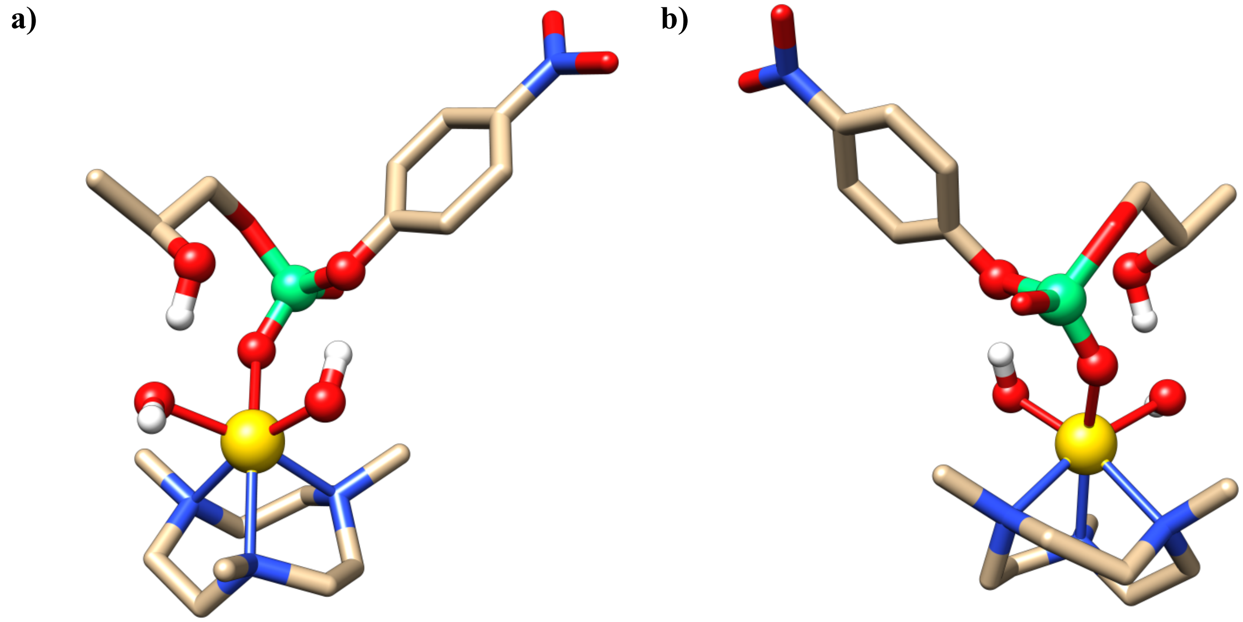

Figure S7. Two different views of the DFT minimization of the structure of the bis-deprotonated active species of complex **2** coordinated to HPNP ([TMTACN-Zn(II)$(OH)_2$(HPNP)]).

4.2.DFT minimized structures

[TACN-Zn(II)(OH)(HPNP)]

Energy = -1814.837096 Hartree

P	-1.65559400	0.82219200	0.68179200
O	-0.20375400	1.03015100	1.05863000
O	1.09681600	1.41660800	-1.98422900
O	-2.40372200	2.23936000	0.51917300
O	-2.54048100	-0.06892100	1.49183900
N	2.61687000	0.11495000	1.62880200
N	1.37188500	-1.71711600	-0.18875200
N	3.63757400	-0.14312900	-0.98543800
C	2.37693100	-1.28277600	2.04930200
C	1.26501600	-1.96823800	1.25974300
C	2.53246200	-2.36407000	-0.83329400
C	3.33948600	-1.39772100	-1.68988900
C	4.56602400	-0.26031400	0.14783900
C	4.02880100	0.44576400	1.38636700
H	2.11940000	-1.32308800	3.11420600
H	3.31240300	-1.84485900	1.94443700
H	1.29167600	-3.04538100	1.48628400
H	0.28627700	-1.59105200	1.58133500
H	3.17104400	-2.80115200	-0.05569100
H	2.20445900	-3.20004000	-1.46205200
H	4.25429700	-1.91195900	-2.02629900
H	2.76461300	-1.13083600	-2.58633700
H	4.74404200	-1.32173000	0.36072600
H	5.54634800	0.16463400	-0.09975600
H	4.66184000	0.19146900	2.25077200
H	4.08440800	1.53214500	1.24470100
Zn	1.53919000	0.48900800	-0.26752200
C	-1.91770600	3.28037700	-0.35162800
H	-1.37831500	2.84842300	-1.20831400
H	-2.81437700	3.78266500	-0.73097700
C	-1.04873900	4.28490100	0.37648500
H	-1.56845700	4.56016600	1.31037000
C	-0.85223500	5.51011600	-0.48610200
H	-0.17198700	6.21387900	0.00464500
H	-0.41654100	5.22830900	-1.45417000
H	-1.80438900	6.02062800	-0.66577700
O	0.24357500	3.76997400	0.68695300
H	0.16319500	2.84050800	0.97938500
H	0.13621200	1.36983800	-2.05411500
O	-1.53411700	0.26149500	-0.87319000
C	-2.53114500	0.09962000	-1.77669300
C	-3.88043400	0.35374800	-1.50568200
C	-2.12107900	-0.33529700	-3.04335800
C	-4.81530400	0.18256700	-2.51275400
H	-4.20280500	0.68269200	-0.52267000
C	-3.05170200	-0.50722800	-4.04814800
H	-1.06530400	-0.53031700	-3.21644700
C	-4.39454300	-0.24078000	-3.77237300
H	-5.86552900	0.37740700	-2.32080800
H	-2.74488400	-0.83904300	-5.03455000
N	-5.37446200	-0.40875600	-4.82000700
O	-4.99787800	-0.79965500	-5.92463700
O	-6.55293900	-0.15355300	-4.57419100
H	3.98962100	0.52814100	-1.66186700
H	0.50995300	-2.03370500	-0.62684900
H	2.24124000	0.74717700	2.33024000

[TMTACN-Zn(II)(OH)(H_2O)(HPNP)]

Energy = -2009.080818 Hartree

P	-1.72159600	0.62047500	0.28791600
O	-0.26468500	0.73857000	0.64150900
O	1.01295100	0.23801300	-2.30145400
O	-2.52446800	1.98302400	0.56747300
O	-2.54708200	-0.49267400	0.85838900
N	2.88132200	0.14441500	1.91955600
N	1.71126300	-1.86208400	0.19069100
N	3.94532100	-0.27898200	-0.75088700
C	2.72440100	-1.24841900	2.38605900
C	1.61048700	-1.98279900	1.65462600
C	2.92338900	-2.51448900	-0.35188600
C	3.70251900	-1.61870400	-1.30236900
C	4.83269400	-0.29939700	0.43139700
C	4.27178100	0.49761500	1.60031000
H	2.49999100	-1.26358700	3.46207000
H	3.67615600	-1.78074200	2.28089900
H	1.61297900	-3.04383400	1.96065000
H	0.63862000	-1.56113200	1.94524600
H	3.55949400	-2.84553600	0.47632600
H	2.64596300	-3.43156300	-0.89003500
H	4.65340100	-2.11607600	-1.56513100
H	3.13933100	-1.48823000	-2.23530600
H	5.02119800	-1.33754700	0.72653300
H	5.81788400	0.11226600	0.17121100
H	4.92580900	0.35686700	2.47939000
H	4.28146100	1.56805400	1.35390200
Zn	1.84029200	0.40945900	-0.10962100
H	1.27923300	1.10244100	-2.64518800
C	-2.36501600	3.26974100	-0.05989000
H	-2.75943000	3.21809500	-1.08601800
H	-3.01633500	3.92696000	0.52477900
C	-0.95655000	3.83046100	-0.09769800
H	-0.43777300	3.54846300	0.83473100
C	-1.02362700	5.34338900	-0.20391900
H	-0.01464200	5.75712100	-0.30787400
H	-1.60590300	5.63906800	-1.08762000
H	-1.49082800	5.78851400	0.68235800
O	-0.25951600	3.29454700	-1.21160500
H	0.64691200	2.98790900	-0.90749800
O	2.03625700	2.43841600	-0.29083700
H	2.79240100	2.63669400	-0.85372400
H	0.07052700	0.34328800	-2.07840300
C	4.47019400	0.61611200	-1.78137500
H	5.40952700	0.22962800	-2.21260100
H	3.73808600	0.73667800	-2.58833500
H	4.67405900	1.60103600	-1.34597900
C	0.51060700	-2.40544400	-0.44926900
H	0.39431600	-3.48103400	-0.23453500
H	-0.38264000	-1.88471400	-0.08635400
H	0.57431400	-2.27062400	-1.53521000
C	2.31764800	1.08374100	2.88972600
H	2.88359900	1.07111200	3.83689500
H	2.33750600	2.10264200	2.48648300
H	1.27638700	0.81771600	3.10412900
O	-1.70583000	0.49097400	-1.38089100
C	-2.77647300	0.35562700	-2.20355600
C	-4.09841300	0.55530200	-1.78891000
C	-2.48392300	0.02266000	-3.53208000
C	-5.12366100	0.42207800	-2.70925700
H	-4.32865300	0.82363400	-0.76263200
C	-3.50484400	-0.11153000	-4.45145400
H	-1.44741300	-0.12546200	-3.82428500
C	-4.82016900	0.08947700	-4.02861600
H	-6.15398500	0.57860200	-2.40666700
H	-3.28968500	-0.36750100	-5.48398300
N	-5.89315500	-0.04801100	-4.98474200
O	-5.61517100	-0.35276600	-6.14432400
O	-7.04937700	0.14278300	-4.60919500

[TMTACN-Zn(II)$(OH)_2$(HPNP)]

Energy = -2008.595020 Hartree

P	-1.66531700	0.65526500	0.27388300
O	-0.19566700	0.73926100	0.57441600
O	1.26889200	0.37108900	-2.34155500
O	-2.43280900	2.03292800	0.59902700
O	-2.49028000	-0.44380500	0.87603000
N	2.74246400	0.14692100	1.87705400
N	1.70947000	-1.85343500	0.03878900
N	4.03984000	-0.27991100	-0.69744700
C	2.53860400	-1.24758500	2.30956700
C	1.48198900	-1.96655400	1.48667800
C	2.96104000	-2.50939700	-0.39217100
C	3.82710200	-1.61669500	-1.26831900
C	4.81298500	-0.30924500	0.55941300
C	4.15597200	0.48543400	1.67750800
H	2.23558900	-1.27859300	3.36658300
H	3.48900200	-1.79120000	2.26686600
H	1.44719100	-3.02757600	1.79600900
H	0.49207600	-1.53572900	1.69221500
H	3.52222400	-2.84483800	0.48759800
H	2.73258900	-3.42642400	-0.95441500
H	4.79166600	-2.12432800	-1.45431700
H	3.34330600	-1.47918000	-2.24452200
H	4.96861100	-1.34890100	0.86859300
H	5.82130900	0.09776400	0.39370100
H	4.73428200	0.33362700	2.60846100
H	4.19981200	1.55770800	1.44252000
Zn	1.85217100	0.47026300	-0.36123700
C	-2.27826500	3.30882300	-0.04883800
H	-2.67351000	3.24194700	-1.07416200
H	-2.93036200	3.97499000	0.52519300
C	-0.86987500	3.87116200	-0.09053900
H	-0.35959600	3.61513200	0.85495500
C	-0.93502200	5.38129900	-0.23666500
H	0.07614200	5.79068200	-0.33848200
H	-1.50396100	5.65378100	-1.13641800
H	-1.41368700	5.85239300	0.62994500
O	-0.16112400	3.30911600	-1.18191400
H	0.74470900	3.01258200	-0.85896200
O	2.18103500	2.54500800	-0.26395300
H	2.77593700	2.72657400	-1.00050300
H	0.30917300	0.45728400	-2.28456900
C	4.67080500	0.59917700	-1.68020000
H	5.64250200	0.19991800	-2.01999900
H	4.01736700	0.71781700	-2.55202700
H	4.84011200	1.58805700	-1.23913000
C	0.56487200	-2.40340700	-0.68639400
H	0.39378200	-3.46329400	-0.42827200
H	-0.34297500	-1.83724700	-0.44641100
H	0.73688200	-2.33251500	-1.76586200
C	2.13217000	1.07437900	2.82632500
H	2.65982500	1.06343400	3.79682200
H	2.15349300	2.09613000	2.43078300
H	1.08697000	0.79813700	2.99941700
O	-1.71789700	0.54517300	-1.38710800
C	-2.81679500	0.36589000	-2.15446200
C	-4.12661500	0.53111100	-1.68692000
C	-2.57035300	0.01695000	-3.48902100
C	-5.18572300	0.33983200	-2.55690200
H	-4.32065500	0.81026500	-0.65606400
C	-3.62491900	-0.17221700	-4.35907000
H	-1.54128000	-0.10151400	-3.81961600
C	-4.92762300	-0.00990900	-3.88187500
H	-6.20691000	0.46474500	-2.21133300
H	-3.44814800	-0.44293700	-5.39514700
N	-6.03504400	-0.20532700	-4.78472200
O	-5.79863600	-0.51387700	-5.95294200
O	-7.18071300	-0.05806300	-4.35865900

1. Brown, D. A.; Usher, D. A. *J. Chem. Soc.* **1965**, 6558-6564.

2. Frisch, M. J.; Trucks, G. W.; Schlegel, H. B.; Scuseria, G. E.; Robb, M. A.; Cheeseman, J. R.; Scalmani, G.; Barone, V.; Mennucci, B.; Petersson, G. A.; Nakatsuji, H.; Caricato, M.; Li, X.; Hratchian, H. P.; Izmaylov, A. F.; Bloino, J.; Zheng, G.; Sonnenberg, J. L.; Hada, M.; Ehara, M.; Toyota, K.; Fukuda, R.; Hasegawa, J.; Ishida, M.; Nakajima, T.; Honda, Y.; Kitao, O.; Nakai, H.; Vreven, T.; Montgomery, J. A., Jr.; Peralta, J. E.; Ogliaro, F.; Bearpark, M.; Heyd, J. J.; Brothers, E.; Kudin, K. N.; Staroverov, V. N.; Kobayashi, R.; Normand, J.; Raghavachari, K.; Rendell, A.; Burant, J. C.; Iyengar, S. S.; Tomasi, J.; Cossi, M.; Rega, N.; Millam, J. M.; Klene, M.; Knox, J. E.; Cross, J. B.; Bakken, V.; Adamo, C.; Jaramillo, J.; Gomperts, R.; Stratmann, R. E.; Yazyev, O.; Austin, A. J.; Cammi, R.; Pomelli, C.; Ochterski, J. W.; Martin, R. L.; Morokuma, K.; Zakrzewski, V. G.; Voth, G. A.; Salvador, P.; Dannenberg, J. J.; Dapprich, S.; Daniels, A. D.; Farkas, Ö. ; Foresman, J. B.; Ortiz, J. V.; Cioslowski, J.; Fox, D. J. Gaussian 09 , revision 7.0; Gaussian, Inc.: Wallingford, CT, **2009**.

3. Zhao, Y.; Truhlar, D.G. The M06 suite of density functional for main group thermochemistry, thermochemical kinetics, non-covalent interactions, excited states, and transition elements: two new functional and systematic testing of four M06-class functional and 12 other function. *Theor. Chem. Acc.* **2007**, *120*, 215-241.

4. Cossi, M.; Barone, V. Time-dependent density functional theory for molecules in liquid solutions. *J. Chem. Phys.* **2001**, *115*, 4708.

5. Cossi, M.; Rega, N.; Scalmani, G.; Barone, V. Energies, Structures and Electronic Properties of Molecules in Solution with the C-PCM Solvatation Model. *J. Comput. Chem.* **2003**, *24*, 669-681.

6. Hay, P. J.; Wadt, W. R. Ab initio effective core potentials for molecular calculations. Potentials for the transition metal atoms Sc to Hg. *J. Chem. Phys.* **1985**, *82*, 270.

7. McLean, A. D.; Chandler, G. S. Contracted Gaussian basis sets for molecular calculations. I. Second row atoms, Z=11-18. *J. Chem. Phys.* **1980**, *72*, 5639.
